# An observational study of initial HIV RNA decay following initiation of combination antiretroviral treatment during pregnancy

**DOI:** 10.1186/s12981-020-00297-w

**Published:** 2020-07-13

**Authors:** Jasmini Alagaratnam, Helen Peters, Kate Francis, Natasha Kay, Yvonne Gilleece, Fionnuala P. Finnerty, Rosanna E. Grimes, Sarah Parry, Mags Portman, Brenton C. Wait, Rimi Shah, Sherie Roedling, David A. Hawkins, Sarah Chitty, Liat Sarner, Rebecca Marcus, Anna Hartley, Achyuta V. Nori, Melanie Rosenvinge, Graham P. Taylor, the London HIV Perinatal Research Group

**Affiliations:** 1 Section of Virology, Department of Infectious Disease, Faculty of Medicine, Imperial College London, London, W2 1PG UK; 2 Department of Genitourinary Medicine, St Mary’s Hospital, Imperial College Healthcare NHS Trust, London, W2 1NY UK; 3 Clinical Trials Centre, Ground Floor, Winston Churchill Wing, St Mary’s Hospital, Praed Street, London, W2 1NY UK; 4 National Surveillance of HIV in Pregnancy and Childhood, UCL Great Ormond Street Institute of Child Health, 30 Guildford Street, Holborn, London, WC1N 1EH UK; 5 The Holborn Medical Centre, 64-66 Lamb’s Conduit Street, Holborn, London, WC1N 3NA UK; 6 Royal Sussex County Hospital, Eastern Road, Brighton, BN2 5BE UK; 7 Homerton University Hospital Foundation Trust, Homerton Row, Clapton, London, E9 6SR UK; 8 Royal Free Hospital, Pond Street, London, NW3 2QG UK; 9 Mortimer Market Centre, Capper Street, Bloomsbury, London, WC1E 6JB UK; 10 Chelsea and Westminster Hospital NHS Foundation Trust, 369 Fulham Road, Chelseas, London, SW10 9NH UK; 11 The Royal London Hospital, Whitechapel Road, Whitechapel, London, E1 1FR UK; 12 Guy’s and St Thomas’ NHS Foundation Trust, Westminster Bridge Road, London, SE1 7EH UK; 13 University Hospital Lewisham, Lewisham High Street, London, SE13 6LH UK

**Keywords:** Antiretroviral therapy, Monitoring, Novel observations, Treatment outcomes

## Abstract

**Background:**

In pregnancy, reduction of HIV plasma viral load (pVL) for the prevention of vertical transmission is time-constrained. The study primary objective is to investigate factors associated with faster initial HIV RNA half-life decay when combination antiretroviral treatment (cART) is initiated in pregnancy.

**Methods:**

This was a multicentre, retrospective, observational study, conducted in south England, United Kingdom, between August 2001 and February 2018. Data were extracted from case notes of eligible women initiating cART during the index pregnancy. Anonymised data were collated and analysed centrally. Regression analyses were conducted to determine factors associated with faster HIV RNA half-life decay in the first 14 days after commencing cART (first-phase), and with achieving an undetectable maternal pVL by 36 weeks’ gestation. We then assessed whether HIV- and obstetric- related parameters differed by antiretroviral third agent class and whether the proportions of women with undetectable pVL at 36 weeks’ gestation and at delivery differed by antiretroviral third agent class.

**Results:**

Baseline pVL was the only independent factor associated with faster first-phase HIV RNA half-life decay on commencing cART. Lower pVL on day 14 after starting cART was associated with an increased likelihood of achieving an undetectable pVL by 36 weeks’ gestation. Integrase inhibitor-based cART was associated with a faster first-phase HIV RNA half-life decay on commencing cART. Overall, 73% and 85% of women had an undetectable pVL at 36 weeks’ gestation and at delivery respectively, with no significant difference by antiretroviral third agent class.

**Conclusions:**

Only high baseline pVL independently contributed to a faster rate of first-phase viral half-life decay. pVL at 14 days after initiating cART allows early identification of treatment failure. In the first 14 days after initiating cART in pregnancy, integrase inhibitor-based cART reduced maternal pVL faster than protease inhibitor- and non-nucleoside reverse transcriptase-based cART. While our study findings support INSTI use when initiated in pregnancy especially when initiated at later gestations and in those with higher baseline pVL, other non-INSTI based cART with more data on safety in pregnancy also performed well.

## Background

In the UK, opt-out antenatal HIV-1/2 (hereafter referred to as HIV) screening, combination antiretroviral treatment (cART) and infant formula feeding have successfully reduced rates of vertical transmission of HIV from 25% in 1993 [1], to the present national rate of < 0.5% [2–4]. In recent years, the majority of pregnant women living with HIV (WLWH) in the UK conceive on cART and maintain HIV suppression throughout pregnancy [4]. However, as recently as 2015–2016, 30% of pregnant WLWH in the UK were still initiating cART during pregnancy [5].

Guidance regarding the timing of cART initiation and the class of compounds used during pregnancy has evolved over the study period and continue to vary between guidelines [6–9]. British HIV Association (BHIVA) guidelines recommend that if maternal plasma viral load (pVL) is > 100,000 copies/mL and/or CD4+ count is < 200 cells/µL, cART should be initiated within the first trimester, and that all women should have commenced cART by 24 weeks’ gestation [10]. Women presenting with untreated HIV beyond 28 weeks’ gestation should initiate cART immediately, and if pVL is > 100,000 copies/mL, a three- or four-drug regimen including raltegravir or dolutegravir is recommended. Read et al. demonstrated that delaying the initiation of cART beyond 26.3 weeks’ gestation when baseline pVL is < 10,000 copies/mL, or beyond 20.4 weeks’ gestation when baseline pVL is > 100,000 copies/mL, was associated with a reduced likelihood of achieving pVL < 50 copies/mL by delivery [11].

The rationale behind recommending the initiation of integrase strand transferase inhibitor (INSTI)-based cART, especially later in pregnancy, is the presumption that a more rapid reduction in maternal pVL will result in a higher chance of having an undetectable maternal pVL by delivery, with subsequent lower rates of perinatal HIV acquisition. Maternal viral burden is a strong predictor of perinatal HIV transmission, with a vertical transmission risk of 63% when untreated maternal pVL is > 100,000 copies/mL during pregnancy and at delivery [12], compared to 0.1% when maternal pVL is < 50 copies/mL on cART [2]. The risk of vertical transmission increases with shorter periods on cART prior to delivery: 3.5% with > 16 weeks of cART and 9% in women who initiate cART < 4 weeks prior to delivery [13]. However, the reductions in HIV vertical transmission are only partly explained by reductions in maternal pVL: firstly, in ACTG 076, maternal pVL at delivery in women initiating zidovudine monotherapy during pregnancy was only 0.24 log_10_ HIV RNA copies/mL lower than in the placebo group, but HIV vertical transmission reduced by 67% in the zidovudine group [14, 15]. Secondly, a single-dose of nevirapine administered at the onset of labour was capable of reducing HIV vertical transmission by nearly 50% [16] compared to zidovudine monotherapy initiated during labour. Given these findings, the reduction in vertical transmission in WLWH on cART during pregnancy can be considered to be the result of both treatment as prevention and infant peri-exposure prophylaxis.

Observational studies suggest that in the absence of cART and in exclusively formula-fed babies, two-thirds of HIV vertical transmissions occur during the intrapartum period [17]. However, in the cART era, 80% of HIV vertical transmission is thought to occur during the later stages of the antepartum period, prior to the onset of labour [18]. For women with untreated HIV infection, strategies that rapidly suppress maternal pVL during pregnancy are desirable, although the evidence that this directly translates into fewer HIV vertical transmissions is not yet available.

Modelling and clinical studies have demonstrated that when three or more antiretroviral drugs are initiated, a rapid exponential decline in pVL occurs in the first 6–14 days [19, 20], followed by a slower, second phase exponential decline, before pVL suppression [19, 21]. Choice of cART has been shown to affect time to viral suppression, notably with INSTI-based regimens achieving viral suppression significantly faster than other drug classes [22, 23].

The primary aim of our study was to investigate the factors associated with a faster first-phase plasma HIV RNA decay in women initiating cART during pregnancy. Secondary aims included investigating factors associated with an increased likelihood of having an undetectable pVL by 36 weeks’ gestation (given that in the UK, recommendations for mode of delivery are guided by maternal pVL at 36 weeks’ gestation [10] and investigating differences in HIV- and obstetric-related parameters when stratified by antiretroviral third agent class.

## Methods

### Study setting and ethical considerations

We conducted a retrospective case note review of all women initiating cART in pregnancy at HIV tertiary centres in south England, United Kingdom. The overall study was conducted between August 2001 and February 2018 to avoid bias, but the actual start dates varied according to site. All data were obtained as part of routine clinical care, anonymised at site, and then collated centrally for analysis. As per the National Research Ethics Service guidelines [24], additional patient consent and ethical approval were not required.

### Eligibility criteria

Inclusion criteria were any woman commencing or re-commencing cART during the index pregnancy with a quantifiable pVL at baseline, and further evaluation on day 14 (± 3 days) following cART initiation. Subjects were excluded if poor adherence to cART was reported by either the subject or clinician.

### Data collection

Clinical data collected included baseline demographics, HIV-specific parameters (cART history, CD4+ T cell count and pVL during index pregnancy) and obstetric and infant specific information (estimated due date and actual delivery date). Aggregate level data on infant HIV infection status (where available) was provided by the National Surveillance of HIV in Pregnancy and Childhood [25].

### Outcome measures

The primary outcome was first-phase plasma HIV RNA half-life decay rate, 14 days after commencing cART. First phase HIV RNA half-life decay (T/2) was calculated using the formula: *n* × log_10_ 0.5/log_10_ (VL_1_/VL_BL_), where *n* = number of days to VL_1_; VL_1_ = plasma HIV RNA load measured 14 days following the initiation of cART and VL_BL_ = plasma HIV RNA load measured prior to the initiation of cART. T/2 was only calculated where VL_1_ was quantifiable. In a secondary analysis, when VL_1_ was reported as < 50 or < 20 HIV RNA copies/mL at day 14, a value of 49 or 19 HIV RNA copies/mL were imputed respectively, to enable an estimation of the slowest first phase T/2 in these women. Secondary outcomes were time taken to achieve undetectable maternal pVL (lower than the limits of quantification) and proportion of women with an undetectable pVL by 36 weeks’ gestation and by delivery. Subjects who did not achieve an undetectable pVL were censored at delivery.

### Statistical analyses

Descriptive statistics summarised variables using median (interquartile range) and total (percentage). For the purposes of this study, the impact of the three dual nucleos(t)ide reverse transcriptase inhibitors (NRTIs) used were considered equal. In subjects on quadruple therapy comprising dual NRTIs, an INSTI and a protease inhibitor (PI), T/2 was presumed to reflect the INSTI component.

Maternal pVL of < 50 copies/mL were regarded as undetectable, including those measured between 20 and 49 copies/mL. All quantifiable pVL ≥ 20 copies/mL were included when calculating T/2. Time taken to achieve an undetectable pVL was calculated assuming that the date an undetectable pVL was achieved was the mid-point between the last detectable and first undetectable pVL.

Regression analysis was performed to investigate factors associated with faster T/2 (linear regression analysis) and with having an undetectable pVL at 36 weeks’ gestation (logistic regression analysis). Kruskall-Wallis test was used to assess whether HIV- and obstetric- related parameters differed by antiretroviral third agent class. Mann–Whitney test (with Bonferroni correction) was then used to compare the parameters between antiretroviral third agent class. Fisher’s exact test was used to assess whether the proportions of women with undetectable pVL at 36 weeks’ gestation and at delivery differed by antiretroviral third agent class. All statistical analyses were conducted using SPSS version 24 (IBM Corp, Armonk, NY, US). Statistical significance was defined as < 0.05 throughout.

## Results

Of the 221 pregnant women at 11 participating HIV centres who initiated cART during the index pregnancy and had sufficient data for analysis, 192 had a detectable pVL after 14 days therapy. 29 women had VL_1_ < 50 copies/mL and are described separately.

### Baseline characteristics

Baseline characteristics of the 192 women included in the main analysis are described in Table 1. 83.3% were Black African/Caribbean, 98.5% identified heterosexual intercourse as their route of HIV acquisition, 63.5% were cART-naïve and median CD4^+^ count prior to initiating cART (baseline) was 339 cells/µL (Table 1). 126 (65.6%) had pre-cART (baseline) plasma HIV RNA < 30,000 copies/mL, 37 (19.3%) between 30,000 and 100,000 HIV RNA copies/mL and 29 (15.1%) had baseline plasma HIV RNA > 100,000 copies/mL. 91% of women commenced cART before 28 weeks’ gestation: 181 (94.3%) women initiated a dual NRTI backbone plus a third agent, 11 (5.7%) initiated abacavir, lamivudine and zidovudine (ABC/3TC/AZT) (Table 1) and seven (3.6%) initiated quadruple cART. Amongst the 192 women included in the main analysis, pVL fell by a median of 1.8 log_10_ HIV RNA copies/mL 14 days after initiating cART, and median T/2 was 2.5 days (Table 1).

**Table 1:** Baseline characteristics, cART regimens and responses of the women who commenced cART during pregnancy

Values are median (IQR) or total (%)	Women initiating cART during index pregnancy and with a quantifiable HIV RNA at day 14 (*n* = 192)	Women initiating cART during index pregnancy and with HIV RNA below the limits of quantification at day 14 (*n* = 29)
Age (years)	31.0 (26.5, 34.0)	31.0 (25.5, 36.0)
Black African and/or Caribbean ethnicity	160 (83.3)	26 (89.7)
HIV acquired via heterosexual intercourse	189 (98.5)	28 (96.6)
Hepatitis B and/or C co-infection	2 (1.0)	4 (13.7)
Received cART previously	70 (36.5)	9 (31.0)
Gestational age when cART initiated, weeks	20.8 (16.9, 24.4)	21.9 (19.0, 24.3)
Baseline CD4^+^ T-cell count, cells/µL	339 (212, 481)	520 (334–637)
Baseline plasma HIV RNA, log_10_ copies/mL	4.2 (3.8, 4.7)	3.1 (2.9–3.6)
Plasma HIV RNA 14 days after initiating cART, log_10_ copies/mL	2.4 (2.1, 2.9)	All < 1.7
First-phase HIV RNA half-life decay, days	2.5 (2.1, 3.0)	2.9 (2.2, 3.6)
Women with pVL < 50 copies/mL at 36 weeks’ gestation	139 (72.4)	29 (100)
Women with pVL < 50 copies/mL at delivery	163 (84.9)	29 (100)
Gestational age at delivery, weeks	39.1 (38.0, 40.4)	39.0 (38.0, 40.0)
cART regimen used		
ABC/3TC/AZT	11 (5.7)	3 (10.3)
Dual NRTI backbone	181 (94.3)	26 (89.7)
AZT + 3TC	75 (39.1)	11 (37.9)
ABC + 3TC	41 (21.4)	6 (20.7)
TDF + FTC	65 (33.9)	9 (31.0)
Dual NRTI backbone + PI	132 (68.8)	20 (69.0)
Atazanavir/ritonavir	60 (31.1)	9 (31.0)
Darunavir/ritonavir	11 (5.7)	1 (3.4)
Lopinavir/ritonavir	50 (26.0)	3 (10.3)
Saquinavir/ritonavir	11 (5.7)	7 (24.1)
Dual NRTI backbone + NNRTI	32 (16.7)	2 (6.9)
Nevirapine	26 (13.5)	2 (6.9)
Efavirenz	5 (2.6)	0
Rilpivirine	1 (0.5)	0
Dual NRTI backbone + INSTI	17 (8.9)	4 (13.8)
Raltegravir	11 (5.7)	1 (3.4)
Dolutegravir	6 (3.1)	3 (10.3)
Infant HIV status at birth		
HIV negative	150 (78.1)	21 (72.4)
Data not available	42 (21.9)	8 (27.6)

Baseline characteristics, antiretroviral treatment regimens and responses of the 221 women living with HIV, who commenced combination antiretroviral therapy during index pregnancy

*IQR* interquartile range, *cART* combination antiretroviral treatment, *ABC* abacavir, *3TC* lamivudine, *AZT* zidovudine, *TDF* tenofovir disoproxil fumarate, *FTC* emtricitabine, *PI* protease inhibitor, *NNRTI* non-nucleoside reverse transcriptase inhibitor, *INSTI* integrase strand transferase inhibitor

### Factors associated with first-phase plasma HIV RNA half-life decay

Univariate linear regression analysis demonstrated that lower baseline CD4+ T-cell count, higher VL_BL_ and INSTI-based cART were associated with faster viral decay (shorter T/2) (Table 2). However, in multivariable regression analysis, only higher VL_BL_ remained independently associated with shorter T/2 (− 0.61 days per 1 log_10_ plasma HIV RNA higher, p < 0.001) (Table 2).

**Table 2:** Linear regression analysis investigating factors associated with first-phase HIV RNA half-life decay

	Univariate analysis		Multivariate analysis	
	Change (95% CI)^a^	*p*-value	Change (95% CI)^a^	*p*-value
Maternal age, per 1-year higher	0.02 (− 0.01, 0.05)	0.24		
Black African/Caribbean ethnicity	− 0.13 (− 0.61, 0.35)	0.17		
HIV acquired via heterosexual intercourse	1.08 (− 0.34, 2.51)	0.14		
Previous cART use	− 0.08 (− 0.45, 0.29)	0.67		
Baseline CD4^+^ T-cell count, per 10 cells/µL increase	0.01 (0.00, 0.02)	0.003	0.00 (− 0.01, 0.01)	0.47
Baseline plasma HIV RNA, per 1 log_10_ copies/mL higher	− 0.71 (− 0.97, − 0.46)	< 0.001	− 0.611 (− 0.918, − 0.305)	< 0.001
Gestational age when cART was started, per 1-week higher	− 0.02 (− 0.05, 0.01)	0.21		
Antiretroviral third agent class				
PI	0.34 (− 0.04, 0.72)	0.08		
NNRTI	− 0.27 (− 0.74, 0.21)	0.27		
INSTI	− 0.84 (− 1.46, − 0.23)	0.007	− 0.365 (− 0.982, 0.251)	0.24

*CI* confidence interval, *cART* combination antiretroviral treatment, *PI* protease inhibitor, *NNRTI* non-nucleoside reverse transcriptase inhibitor, *INSTI* integrase strand transferase inhibitor

^a^Change (95% CI) reflects the associated impact of each independent variable in the model on the first-phase HIV RNA half-life decay

### Factors associated with having an undetectable maternal plasma viral load at 36 weeks’ gestation

In multivariate logistic regression analysis, the following factors were associated with a higher probability of having an undetectable maternal pVL by 36 weeks’ gestation: lower maternal pVL 14 days after initiating cART (odds ratio (OR) 0.05, 95% confidence interval (CI) 0.01, 0.19) and earlier gestational age when cART was initiated (OR 0.66, 95% CI 0.58, 0.76) (Table 3).

**Table 3:** Logistic regression investigating factors associated with having an undetectable maternal pVL by 36 weeks’ gestation

	Odds ratio (95% CI)
Maternal age, years	0.99 (0.90, 1.08)
Baseline CD4^+^ T-cell count, cells/µL	1.00 (0.99, 1.05)
Baseline plasma HIV RNA, log_10_ copies/mL	0.88 (0.28, 2.81)
Plasma HIV RNA 14 days after initiating cART, log_10_ copies/mL	0.05 (0.01, 0.19)
Gestational age when cART was started, weeks	0.66 (0.58, 0.76)

Multivariate logistic regression analysis investigating factors associated with having an undetectable maternal plasma viral load by 36 weeks’ gestation

*CI* confidence interval, *cART* combination antiretroviral treatment, *pVL* plasma viral load

### Differences in HIV- and obstetric-related parameters when stratified according to antiretroviral third agent class

T/2 was significantly shorter in women commenced on INSTIs compared to those commenced on PIs, non-nucleoside reverse transcriptase inhibitor (NNRTIs), and ABC/3TC/AZT (p < 0.001, p < 0.001 and p = 0.03, respectively) (Table 4). T/2 was significantly longer in women commenced on PIs compared to NNRTIs, p = 0.03 (Table 4).

**Table 4:** HIV- and obstetric-related parameters, stratified according to antiretroviral third agent class

	PI *n* = 132	NNRTI *n *= 32	INSTI *n *= 17	ABC/3TC/AZT *n *=11	p-values (with Bonferroni correction)
HIV RNA half-life decay, days	2.6 (1.6–11.5)	2.3 (1.4–5.3)	1.8 (1.1–6.0)	2.6 (1.8–10.3)	χ^2^(3) = 30.92, *p *< 0.001 PI vs NNRTI, *p *= 0.03 PI vs INSTI, *p *< 0.001 PI vs ABC/3TC/AZT, *p *= 0.87 NNRTI vs INSTI, *p *< 0.001 NNRTI vs ABC/3TC/AZT, *p *= 0.32 INSTI vs ABC/3TC/AZT, *p *= 0.03
Baseline pVL, log_10_ copies/mL	4.2 (2.5–5.8)	4.3 (2.8–5.4)	5.0 (4.0–5.8)	3.8 (2.9–4.5)	χ^2^(3) = 28.14, *p *< 0.001 PI vs NNRTI, *p *= 0.13 PI vs INSTI, *p *< 0.001 PI vs ABC/3TC/AZT, *p *= 0.04 NNRTI vs INSTI, *p *= 0.18 NNRTI vs ABC/3TC/AZT, *p *= 0.03 INSTI vs ABC/3TC/AZT, *p *< 0.001
Baseline CD4^+^ T-cell count, cells/µL	359 (63–1136)	207 (30–848)	215 (10–700)	574 (290–1008)	χ^2^(3) = 31.20, *p *< 0.001 PI vs NNRTI, *p *< 0.001 PI vs INSTI, *p *= 0.28 PI vs ABC/3TC/AZT, *p *= 0.03 NNRTI vs INSTI, *p *= 0.56 NNRTI vs ABC/3TC/AZT, *p *< 0.001 INSTI vs ABC/3TC/AZT, *p *= 0.06
Gestational age when cART was started, weeks	21.2 (5.7–39.6)	18.9 (4.9–34.0)	17.6 (8.1–34.4)	21.9 (10.9–24.0)	χ^2^(3) = 3.67, *p *= 0.299
pVL 14 days after cART was initiated, log_10_ HIV RNA copies/mL	2.5 (1.5–4.1)	2.5 (1.8–3.6)	2.4 (1.5–4.3)	1.9 (1.7–2.9)	χ^2^(3) = 6.94, *p *= 0.074
Time taken to reach pVL < 50 copies/mL, days^a^	51.5 (7–174)	51.5 (21–188)	27.5 (6–121)	32.5 (21–63)	χ^2^(3) = 12.99, *p *= 0.005 PI vs NNRTI, *p *= 0.63 PI vs INSTI, *p *= 0.09 PI vs ABC/3TC/AZT, *p *= 0.07 NNRTI vs INSTI, *p *= 0.05 NNRTI vs ABC/3TC/AZT, *p *= 0.35 INSTI vs ABC/3TC/AZT, *p *= 0.45
Women with pVL < 50 copies/mL at 36 weeks’ gestation	95 (72.0)	21 (65.6)	13 (76.5)	11 (100)	*p *= 0.13
Women with pVL < 50 copies/mL at delivery	113 (85.6)	24 (75.0)	16 (94.1)	11 (100)	*p *= 0.17
Values are median (range) or total (%)					

*PI* protease inhibitor, *NNRTI* non-nucleoside reverse transcriptase inhibitor, *INSTI* integrase strand transferase inhibitor, *ABC* abacavir, *3TC* lamivudine, *AZT* zidovudine, *cART* combination antiretroviral treatment, *pVL* plasma viral load

^a^Only women who achieved plasma viral loads lower than the limits of quantification prior to delivery are included

Women who initiated an INSTI had significantly higher VL_BL_ compared to women initiating PIs and ABC/3TC/AZT (p < 0.001 and p < 0.001, respectively), while women commenced on ABC/3TC/AZT had significantly lower VL_BL_ and higher baseline CD4 + counts compared to women commenced on PIs and NNRTIs (Table 4). Women who initiated NNRTIs had lower baseline CD4 compared to women initiated on PIs. While there was no statistically significant difference in the gestational age at which cART was commenced when stratified according to the three-third agent antiretroviral classes and ABC/3TC/AZT (Table 4), higher VL_BL_ was significantly associated with earlier gestational age when cART was initiated, p = 0.02.

Between the four groups, the overall time to an undetectable pVL was numerically shorter in women commenced on INSTIs, but statistically only approached significance when comparing women initiating INSTIs (27.5 days) and NNRTIs (51.5 days), p = 0.05 (Table 4).

An undetectable pVL was seen in 72.9% of all women by 36 weeks gestational age with broadly similar rates across the three antiretroviral third agent classes: PIs 72.0%, NNRTIs 65.6% and INSTIs 76.5% (Table 4). 164 (85.4%) women had an undetectable pVL by delivery: PIs 85.6%, NNRTIs 75.0% and INSTIs 94.1% (Table 4). All women who initiated ABC/3TC/AZT had undetectable pVL at both 36 weeks’ gestation and delivery. Statistically, the proportions of women with undetectable pVL at 36 weeks’ gestation and at delivery did not differ significantly by antiretroviral third agent class (p = 0.13 and p = 0.17, respectively).

### Women with pVL < 50 copies/mL at day 14 following cART initiation

Baseline characteristics for the 29 women with VL_1_ < 50 copies/mL were broadly similar to the 192 women included in the main analysis (Table 1). However, this cohort of 29 women had significantly higher baseline CD4+ count (p = 0.001), lower VL_BL_ (p < 0.001), a higher proportion with Hepatitis B co-infection (p = 0.007), and all women had VL_BL_ < 30,000 copies/mL. The longest estimated T/2 in this cohort of 29 women was similar to the T/2 seen in the subgroup of women included in the main analysis with VL_BL_ < 30,000 copies/mL, p = 0.354.

### Infant HIV infection rates

Infant HIV infection status was established for 171/221. All 171/221 infants were negative by molecular HIV testing at birth. For the remaining 50/221, infection status was not available. Although data on infant HIV peri-exposure prophylaxis were not available, each centre managed infants according to the contemporaneous version of the BHIVA guidelines on the management of HIV infection in pregnancy.

## Discussion

In this study, higher baseline plasma viral load (VL_BL_) was the sole independent predictor of faster first-phase HIV RNA decay. Secondly, lower maternal pVL 14 days after cART initiation and earlier gestational age when cART was initiated independently predicted a higher likelihood of having an undetectable maternal pVL at 36 weeks’ gestation.

Overall, women in this cohort experienced a median 1.8 log_10_ copies/mL decline in pVL 14 days after initiating cART, with the shortest T/2 and thus, largest HIV RNA decline seen in women on INSTIs (despite small numbers of women on INSTIs), in keeping with the DolPHIN-1 (Dolutegravir in pregnant HIV mothers and their neonates) [26] study results demonstrating superior virological responses with dolutegravir-based cART compared to efavirenz-based cART when initiated in late pregnancy in low and middle-income settings. This strengthens the recommendation for assessing response to cART at this early stage and emphasises that clinicians and patients should expect around a 99% reduction in maternal pVL after 2 weeks of cART. Less convincing reductions in pVL at day 14, regardless of third agent in the cART regime, should be a cause for concern. Current BHIVA guidelines recommending all women should have started cART by 24 weeks’ gestation are based on the results published by Read et al. [11]. However, it is important to note that cART regimens in this study only included PIs, NNRTIs and ABC/3TC/AZT and did not include INSTI-based cART. Given the faster rate of HIV RNA declines in women on INSTIs as demonstrated in our study and other published data [26], the latest gestational age before which women on INSTI-based cART should have commenced cART will need to be explored in future studies.

Excluding ABC/3TC/AZT due to small subject numbers, only 66–77% of women on PIs, NNRTIs and INSTIs had undetectable pVL at 36 weeks’ gestation, when obstetric delivery plans are usually finalised. Of these women, those on INSTIs had the highest proportion of undetectable pVL by delivery and a shorter time to an undetectable pVL, despite starting with significantly higher VL_BL_, mirroring the findings seen in randomised trials of cART initiation during pregnancy performed in low and middle-income countries (DolPHIN-2 study [27]: dolutegravir versus efavirenz-based cART and the National Institute of Child Health and Human Development P1081 study [28]: raltegravir versus efavirenz-based cART). At present, INSTIs are considered the preferred antiretroviral third agent for women presenting late in pregnancy, yet in our study cohort, gestational age when cART was initiated was numerically earlier with INSTIs than with the other classes, reflecting the recommendation to initiate cART earlier in women with high baseline pVL. Over 90% of women in our study initiated cART before 28 weeks’ gestation but the low overall maternal CD4+ counts prior to initiating cART indicates that many of these women were presenting late in the course of their HIV infection.

While the small number of women who initiated ABC/3TC/AZT in pregnancy all had an undetectable pVL at delivery, they had lower VL_BL_ and higher CD4+ counts than women who initiated PIs and NNRTIs, consistent with a targeted approach for initiating ABC/3TC/AZT. The results seen in this subgroup of women should therefore not be extrapolated to women with higher VL_BL_ and lower baseline CD4+ counts.

Limitations of our study include the small number of subject initiating INSTIs, preventing statistical analysis of the clinical effects between the individual INSTI agents, and the long data collection period, meaning that data were collected and analysed for antiretroviral drugs that are now rarely used or commenced in the UK. While the exclusion of subjects with poor adherence reported by either the subject or clinician was necessary to enable the accurate calculation of the first-phase HIV RNA half-life decay during treatment, this may have led to inadvertent subject selection bias. Recent modelling studies have since suggested that on INSTI-based cART regimens, the original first phase T/2 described may be replaced by a split first phase (phase 1a and 1b), followed by the second phase which starts at a lower plasma viral load than the start of the second phase with PI and NNRTIs [29]. We were unable to explore this concept in our study.

The findings of this observational study support the use of INSTIs when initiating cART in pregnancy, especially in women presenting later in pregnancy and/or with higher VL_BL_ on the basis of achieving rapid reductions in pVL and an undetectable viral load at delivery. However, it is important to note that non-INSTI-based regimens also performed well. Whilst awaiting further data on the higher incidence (0.3%) of neural tube defects in infants born to women conceiving on dolutegravir [30], there may well be a decrease in the use of dolutegravir, and possibly INSTIs as a whole, especially when initiating cART in the first trimester of pregnancy. In the meantime, other third agent antiretroviral drugs with more data on safety and efficacy in pregnancy that perform well in pregnancy are likely to be used in preference.

## Conclusions

Higher baseline maternal pVL was the sole independent factor associated with faster HIV RNA decay 14 days after cART initiation. Lower maternal pVL 14 days after initiating cART, but not lower baseline maternal pVL, was associated with a higher chance of having an undetectable maternal pVL at 36 weeks’ gestation.

## Data Availability

The datasets generated and analysed during the current study are not publicly available but are available from the corresponding author upon reasonable request.

## Abbreviations

ABC/3TC/AZT: Abacavir/lamivudine/zidovudine
BHIVA: British HIV Association
cART: Combination antiretroviral treatment
DolPHIN study: Dolutegravir in pregnant HIV mothers and their neonates study
INSTI: Integrase strand transferase inhibitor
NRTIs: Nucleos(t)ide reverse transcriptase inhibitors
NNRTIs: Non-nucleoside reverse transcriptase inhibitors
PIs: Protease inhibitors
pVL: Plasma viral load
T/2: First phase HIV RNA half-life decay
VL_1_: Plasma HIV RNA load measured 14 days following the initiation of cART
VL_BL_: Plasma HIV RNA load measured prior to the initiation of cART
WLWH: Women living with HIV
